# Protective Effects of Food-Derived Kaempferol on Pancreatic β-Cells in Type 1 Diabetes Mellitus

**DOI:** 10.3390/foods13233797

**Published:** 2024-11-26

**Authors:** Chenmeng Song, Wei Zheng, Chengyi Song, Houfeng Zhou, Jengyuan Yao

**Affiliations:** 1 School of Public Health, Fujian Medical University, Fuzhou 350005, China; scmyouth@163.com; 2 Department of Pharmacy, Xiamen Medical College, Xiamen 361005, China; 13055482230@163.com (W.Z.); s19141294561@163.com (C.S.); 3 Department of Clinical Medicine, Xiamen Medical University, Xiamen 361005, China; 15205012297@163.com; 4 Key Laboratory of Functional and Clinical Translational Medicine, Fujian Province University, Xiamen Medical College, Xiamen 361005, China

**Keywords:** kaempferol, type 1 diabetes, pancreatic β-cell protection, metabolic disturbances, metabolomics

## Abstract

Background: Kaempferol (KPF), a flavonoid abundant in edible plants, possesses potent anti-inflammatory and antioxidant properties beneficial with notable health benefits. Objective: To evaluate the protective effects of KPF on metabolic disturbances and pancreatic damage in a Type 1 diabetes mellitus (T1DM) mouse model. Methods: Male C57BL/6 mice were divided into normal, T1DM, T1DM + KPF 25 mg/kg, and T1DM + KPF 50 mg/kg groups. T1DM was induced by streptozotocin (STZ). KPF was administered via intraperitoneal injection for 2 weeks. After 4 weeks from the start, metabolic parameters, pancreatic histology, and plasma metabolites were analyzed. Network pharmacology and molecular docking identified key targets and pathways. In vitro, INS-1 cells were used to assess reactive oxygen species (ROS) production and apoptosis. Results: KPF significantly reduced blood glucose (GLU) and triglyceride (TG) levels, increased high-density lipoprotein (HDL) levels, and preserved pancreatic β-cell structure. Metabolomics revealed changes in energy metabolism and oxidative stress-related metabolites. Network analysis highlighted the PI3K/AKT/mTOR pathway, with strong binding affinities to targets such as AKT1. In vitro, KPF decreased ROS production in INS-1 cells; this effect was reversed by a PI3K/AKT inhibitor. KPF also reduced apoptosis in INS-1 cells. Conclusions: KPF ameliorates metabolic disturbances and pancreatic damage in T1DM mice, suggesting potential as a functional food ingredient for diabetes management.

## 1. Introduction

Type 1 diabetes (T1DM) is a metabolic disorder characterized by the autoimmune-mediated destruction of pancreatic β-cells, leading to severe insulin deficiency and chronic hyperglycemia [1]. Commonly diagnosed in children and adolescents, T1DM can also manifest in adults. Despite insulin therapy being the mainstay of treatment, patients remain at risk of long-term complications such as cardiovascular disease, neuropathy, nephropathy, and retinopathy, which significantly affect their quality of life [2]. The disease is marked by persistent hyperglycemia, disrupted energy and lipid metabolism, and structural damage to pancreatic islets due to continuous β-cell loss. Moreover, inflammatory and oxidative stress responses further contribute to tissue damage and disease progression, underscoring the need for therapies that can address these multifaceted metabolic challenges [1].

Lifelong insulin therapy, while essential for managing hyperglycemia, does not fully replicate physiological insulin secretion, often resulting in fluctuations between hyperglycemia and hypoglycemia [3]. Advanced technologies, such as continuous glucose monitoring (CGM), insulin pumps, and artificial pancreas devices, have improved glucose control; however, these options remain costly and are not widely accessible [3,4,5]. Moreover, these treatments do not address the underlying autoimmune destruction of β-cells nor halt disease progression. Immunomodulatory strategies, including islet transplantation and immunosuppressive drugs, offer potential benefits but face challenges such as limited donor availability, immune rejection, and significant side effects [6,7]. Consequently, there remains a need for novel therapies that not only regulate blood glucose but also preserve β-cell function, reduce metabolic disturbances, and mitigate complications associated with T1DM.

Kaempferol (KPF), a flavonoid found in numerous foods and medicinal plants, has gained attention due to its broad range of biological activities, including anti-inflammatory, antioxidant, antiviral, anticancer, cardioprotective, and neuroprotective effects [8,9,10]. It has shown promise in various therapeutic areas, such as neurodegenerative diseases (NDDs), where it can prevent amyloid fibril deposition, inhibit microglia activation, and reduce oxidative stress [8]. In kidney diseases, KPF offers nephroprotective benefits by mitigating oxidative stress and inflammation [9]. Additionally, it has demonstrated cardioprotective effects in cardiac injury models by reducing apoptosis and fibrosis and preserving mitochondrial function [11]. KPF has also been highlighted for its anticancer potential, modulating key signaling pathways involved in tumor growth [10]. Furthermore, KPF’s role in managing metabolic diseases, such as diabetes and non-alcoholic fatty liver disease, through the regulation of pathways such as PI3K/AKT, NF-kB, Nrf2, and AMPK underscores its potential as a multi-target therapeutic agent [12].

Given KPF’s promising pharmacological properties across various disease models—including its anti-inflammatory, antioxidant, and metabolic regulation capabilities—we aimed to evaluate its therapeutic effects in T1DM. T1DM is characterized by hyperglycemia, β-cell dysfunction, and metabolic disturbances, for which current treatments are insufficient to halt disease progression. Recent studies have highlighted KPF’s role in modulating key metabolic pathways, improving glucose metabolism in the liver and muscle, and reducing gluconeogenesis [13,14]. Additionally, KPF has shown potential in promoting insulin and GLP-1 release, offering renoprotective and antifibrotic benefits in diabetic nephropathy [15]. Therefore, we systematically investigated KPF’s impact on T1DM, particularly its ability to preserve β-cell function, regulate glycolipid metabolism, and ameliorate metabolic dysfunction, further exploring its potential as a multi-target natural compound for T1DM management [16].

## 2. Materials and Methods

### 2.1. Materials

KPF was purchased from Solarbio (Cat. No. SK8030, Beijing, China). Streptozotocin (STZ) was obtained from Macklin (Cat. No. S817944, Shanghai, China). Methanol (Cat. No. M433266) and acetonitrile (Cat. No. A800362) were purchased from Aladdin (Shanghai, China) and Macklin, respectively. Formic acid was from Waters (Cat. No. 700002669, Massachusetts, USA). The caspase-9 antibody was obtained from Santa Cruz Biotechnology (Cat. No. sc-56073, Dallas, TX, USA), and the reactive oxygen species (ROS) detection kit was from Beyotime (Cat. No. S0033M, Shanghai, China). A biochemical analyzer (Seamaty Technology, Chengdu, China), a cryostat microtome (Thermo Fisher Scientific, Waltham, MA, USA), and an inverted fluorescence microscope (Leica Microsystems, Wetzlar, Germany) were used. Other reagents included hematoxylin and eosin (H&E) staining reagents and RPMI-1640 medium supplemented with fetal bovine serum (FBS), penicillin, and streptomycin were purchased from Beyotime. Male C57BL/6 mice (6–8 weeks old, specific pathogen-free) were purchased from Fuzhou NORDEN Biotechnology Co., Ltd. (Production License SCXK (ZHE) 2019-0002, Fuzhou, China) and housed under controlled environmental conditions (temperature 18–26 °C, relative humidity 40–60%) with a natural light-dark cycle and food and water provided ad libitum. Specifically, we state that all animal experiments were approved by the Experimental Animal Ethics Committee of Xiamen Medical College (Approval Number: 20240207015, Approval Date: 7 February 2024). Furthermore, the study ensures adherence to ARRIVE guidelines for animal welfare. INS-1 cells were obtained from the National Resource Center for Cell Lines (Beijing, China).

### 2.2. Animal Experiments

Male C57BL/6 mice were randomly divided into four groups (*n* = 10 per group): normal, T1DM, T1DM + KPF25, and T1DM + KPF50. After two weeks of acclimation, T1DM was induced in the T1DM, T1DM + KPF25, and T1DM + KPF50 groups by intraperitoneal injection of STZ at 50 mg/kg in PBS once daily for five consecutive days, following established protocol [17]. The normal group received PBS alone. Simultaneously, KPF was administered via intraperitoneal injection to the T1DM + KPF25 and T1DM + KPF50 groups at doses of 25 mg/kg and 50 mg/kg, respectively, once daily for 14 consecutive days, starting from the first day of STZ injection as described in the previous study [18]. The normal and T1DM groups received the vehicle solution. During the four-week experimental period, body weight, food and water intake, and blood glucose levels were monitored weekly. At the end of the treatment, mice were fasted overnight and anesthetized with isoflurane. Blood was collected via enucleation (eyeball removal), and tissues (pancreas and liver) were excised, rinsed with phosphate-buffered saline (PBS), blotted dry, weighed, and stored at −80 °C. The liver index was calculated as the ratio of liver weight to body weight. Pancreatic tissues were fixed in 4% paraformaldehyde for histological analysis.

### 2.3. Biochemical Analysis

Blood samples were collected into heparinized tubes, and plasma was separated by centrifugation at 1000 rpm for 10 min at 4 °C. Plasma glucose (GLU), triglycerides (TG), and high-density lipoprotein cholesterol (HDL) were measured using a biochemical analyzer to assess glycolipid metabolism. Plasma insulin levels were measured using a commercial ELISA kit (Solarbio, Cat. No. SEKM0141) to calculate the Homeostasis Model Assessment of Beta-cell function (HOMA-β), using the formula: HOMA-β = [20 × fasting insulin (μU/mL)]/[fasting glucose (mmol/L) − 3.5].

### 2.4. Histological Analysis and H&E Staining

Pancreatic tissues were fixed in 4% paraformaldehyde for 24 h, cryoprotected in 30% sucrose solution, and embedded in an optimal cutting temperature (OCT) compound. Sections (8 μm thick) were prepared using a cryostat microscope and stained with H and E to assess pancreatic islet morphology.

### 2.5. Metabolomics Analysis

A 50 μL plasma sample was mixed with 400 μL of methanol, vortexed, sonicated for 2 min, and incubated at 4 °C for 1 h to precipitate proteins. After centrifugation at 15,000× *g* for 10 min at 4 °C, the supernatant (400 μL) was evaporated to dryness under a gentle stream of nitrogen gas. The residue was reconstituted in 100 μL of 50% acetonitrile in water and centrifuged at 15,000× *g* for 10 min at 4 °C. The supernatant was used for HPLC-MS/MS analysis. Metabolomic profiling was performed using a Waters Xevo G2-XS QToF mass spectrometer coupled with an ACQUITY UPLC BEH C18 column. Data were acquired in both positive and negative ion modes. The mass spectrometric conditions included a capillary voltage of 2850 V, a source temperature of 120 °C, and a scan range of 50–1000 m/z. Data processing, including peak extraction, alignment, and normalization, was performed using Progenesis QI software (Tables S1 and S2, https://www.nonlinear.com/progenesis/qi/, accessed on 23 November 2024). Differential metabolites were identified based on *p*-values less than 0.05 and annotated using the Human Metabolome Database (HMDB) and Kyoto Encyclopedia of Genes and Genomes (KEGG) database.

### 2.6. Network Pharmacology Analysis

KPF targets were identified using Traditional Chinese Medicine Systems Pharmacology (TCMSP), PubChem, and SwissTargetPrediction databases. T1DM-associated gene targets were obtained from GeneCards and Online Mendelian Inheritance in Man (OMIM) databases. Overlapping targets were identified using the Venny 2.1 tool, and interaction networks were constructed using Cytoscape version 3.7.0. Core targets were screened based on betweenness centrality, closeness centrality, and degree centrality (Table S4). Gene Ontology (GO) and KEGG pathway enrichment analyses were conducted using the Database for Annotation, Visualization, and Integrated Discovery (DAVID) database (version 6.8) (Table S5). A metabolism-gene interaction network was constructed using the Metscape plugin in Cytoscape to identify key metabolites and proteins (Table S6).

### 2.7. Cell Culture and Staining

INS-1 cells were cultured in RPMI-1640 medium with 10% FBS, 100 IU/mL penicillin, and 100 μg/mL streptomycin at 37 °C in a humidified incubator with 5% CO_2_. Cells were seeded in 12-well plates at a density of 2.5 × 10^5^ cells/mL. KPF was added to the culture medium to reach final concentrations of 20 μM, 40 μM, and 80 μM, and cells were incubated with KPF for 24 h. Preliminary experiments were conducted to determine suitable KPF concentrations; results showed that cell viability remained above 50% when cells were treated with KPF concentrations ranging from 0 to 80 μM. Based on these findings, final treatment concentrations of 20 μM, 40 μM, and 80 μM KPF were selected for further experiments. For staining, cells were fixed with 4% paraformaldehyde for 10 min, permeabilized with 0.2% Triton X-100 for 10 min, and blocked with 5% bovine serum albumin (BSA) for 30 min. Cells were incubated with primary antibodies for 2 h at room temperature, followed by fluorescent secondary antibodies for 1 h and DAPI staining for nuclear visualization. Fluorescence images were captured using an inverted fluorescence microscope. ROS production was measured using a ROS detection kit.

### 2.8. Statistical Analysis

Data were expressed as mean ± standard deviation (SD). Statistical analyses were performed using SPSS version 22.0 software. Comparisons between two groups were conducted using the Student’s *t*-test, while multiple group comparisons were performed using one-way ANOVA, followed by the LSD-t test for pairwise comparisons. A *p*-value < 0.05 was considered statistically significant.

## 3. Results

### 3.1. Effects of KPF on Metabolic Parameters and Pancreatic Morphology in T1DM Mice

**Figure 1:**
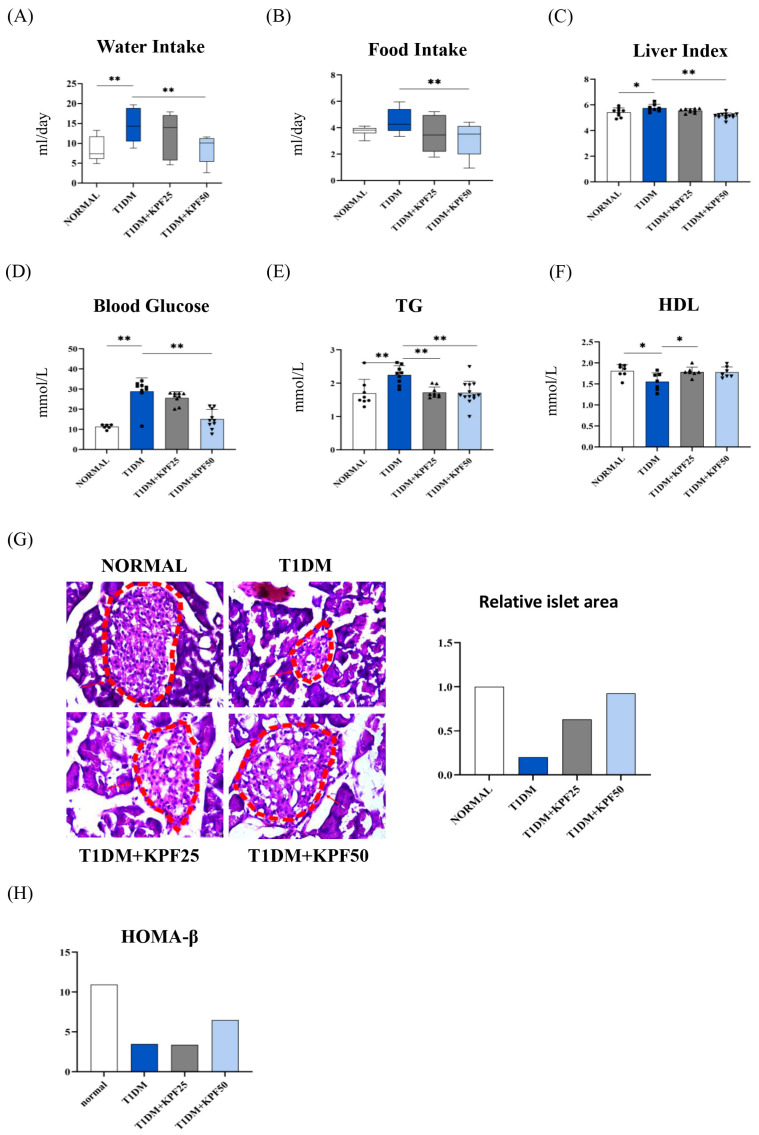
Effects of KPF on metabolic parameters and pancreatic morphology in T1DM Mice. (**A**) water intake, (**B**) food intake, (**C**) liver index, (**D**) blood glucose levels (GLU), (**E**) triglyceride (TG) levels, (**F**) high-density lipoprotein (HDL) levels, and (**G**) representative hematoxylin and eosin (H&E) staining of pancreatic tissue. The relative islet area was quantified, highlighted using arrows in the image, and is presented as a bar chart. Data represent a single measurement and therefore do not include statistical analysis. (**H**) Homeostasis Model Assessment of Beta-cell Function (HOMA-β). Data are presented as mean ± SD. * *p* < 0.05, ** *p* < 0.01 compared to the T1DM group.

Water and food intake were significantly higher in the T1DM group compared to the normal group, whereas the T1DM + KPF50 group showed marked reductions (*p* < 0.01) (Figure 1A,B). The liver index was also significantly increased in the T1DM group compared to the normal group (*p* < 0.05). Treatment with KPF significantly reduced the liver index in the T1DM + KPF50 group (*p* < 0.01), with a trend toward improvement in the T1DM + KPF25 group, though this was not statistically significant (Figure 1C). GLU levels were significantly elevated in the T1DM group compared to the normal group (*p* < 0.05), while KPF treatment in the T1DM + KPF50 group led to a significant reduction (*p* < 0.01) (Figure 1D). TG levels were significantly higher in the T1DM group (*p* < 0.01), whereas both KPF-treated groups showed significant reductions (*p* < 0.01) (Figure 1E). HDL levels were significantly lower in the T1DM group compared to the normal group (*p* < 0.05). KPF treatment increased HDL levels in both treated groups, with a significant increase in the T1DM + KPF25 group (*p* < 0.05) (Figure 1F).

Histological analysis using H and E staining revealed disrupted islet morphology and vacuolated β-cells in the T1DM group. In contrast, both KPF-treated groups exhibited improved islet structure and increased β-cell numbers (Figure 1G). The HOMA-β index, which reflects β-cell function, was reduced in the T1DM group compared to the normal group. KPF treatment, particularly in the T1DM + KPF50 group, showed an improvement in this index, although statistical validation was not performed (Figure 1H).

### 3.2. Metabolic Profiling and Biomarker Analysis of T1DM and KPF-Treated Mice

**Figure 2:**
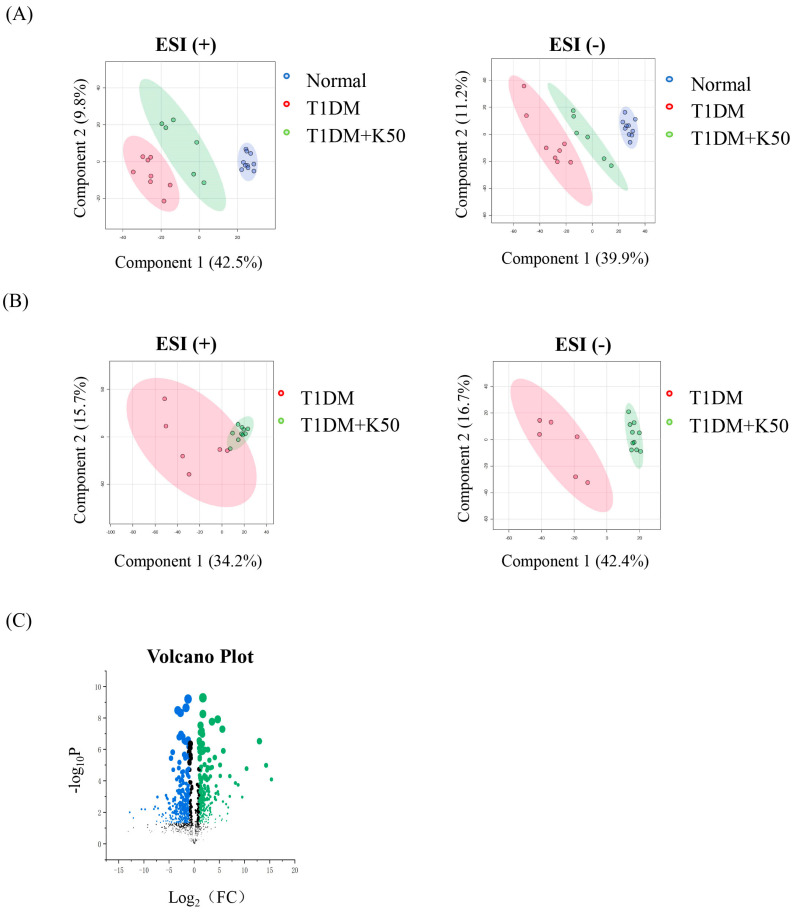
Metabolic profiling and biomarker analysis of T1DM and KPF-treated Mice. (**A**) Principal component analysis (PCA) score plots for normal, T1DM, and T1DM + KPF50 groups in positive and negative ion modes. (**B**) PCA score plots comparing T1DM and T1DM + KPF50 groups. (**C**) Volcano plot showing significantly altered metabolites between T1DM and T1DM + KPF50 groups, with 94 endogenous metabolites identified. Statistical thresholds were set at fold change (FC) > 2 and *p* < 0.05. Blue dots represent significantly downregulated metabolites, and green dots represent significantly upregulated metabolites.

Mass spectrometry data generated 6510 ion signals in positive mode (ESI(+)) and 6385 in negative mode (ESI(−)). These ion signals were analyzed using MetaboAnalyst 6.0. Principal component analysis (PCA) analysis demonstrated distinct separation among normal, T1DM, and T1DM + KPF50 groups in both ion modes, indicating significant differences in metabolite composition (Figure 2A). A separate PCA comparison between the T1DM and T1DM + KPF50 groups also revealed distinct metabolic differences (Figure 2B). Volcano plot analysis identified 7045 significantly altered metabolites between the two groups (Fold Change (FC) > 2, *p* < 0.05), with 94 of these identified as endogenous metabolites in the HMDB database (Figure 2C). Biomarker analysis highlighted the most significant metabolites associated with KPF treatment response, which are summarized in Table 1 (Table S3).

### 3.3. Network Pharmacology Analysis of KPF in T1DM Mice

**Figure 3:**
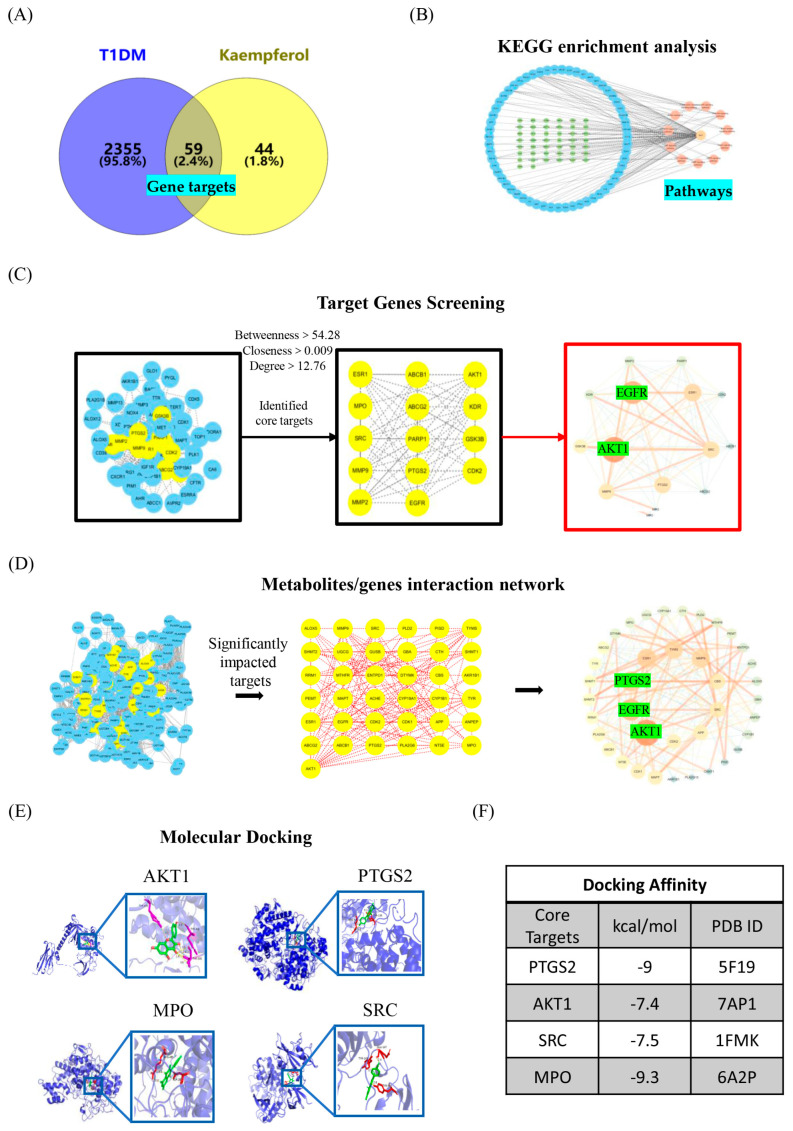
Network pharmacology analysis of KPF in T1DM Mice. (**A**) Venn diagram showing the overlap between KPF drug targets and T1DM-related gene targets. (**B**) Compound-target-pathway network based on KEGG enrichment analysis. (**C**) Visualization of 14 core targets identified by PPI network analysis. (**D**) Metabolite-gene interaction network showing four key targets: PTGS2, AKT1, SRC, and MPO. (**E**) Molecular docking of KPF with these four key targets. (**F**) Binding affinity results of KPF with key targets (kcal/mol).

To explore the therapeutic potential of KPF in T1DM treatment, network pharmacology analysis was conducted. Initial screening using Venny 2.1 identified 59 intersecting targets from 2414 T1DM-related genes and 104 KPF-related drug targets (Figure 3A). These intersecting targets were considered potential targets for KPF in T1DM treatment. A protein-protein interaction (PPI) network was constructed using Cytoscape, and KEGG enrichment analysis was performed to build the compound-target-pathway network (Figure 3B). Based on screening criteria (betweenness centrality > 54.28, closeness centrality > 0.009, degree centrality > 12.76), 14 core targets were identified, including AKT1, EGFR, ESR1, SRC, PTGS2, MMP9, GSK3B, KDR, MMP2, PARP1, CDK2, ABCB1, ABCG2, and MPO, visualized in Figure 3C.

Metabolomic analysis revealed 94 significantly altered endogenous metabolites (Figure 2C). By intersecting these metabolites with 172 identified targets and 59 potential targets from network pharmacology, 40 core targets were identified, including AKT1. The resulting network comprised 40 nodes and 179 edges, and KEGG enrichment identified the top 10 pathways (Table 2). Further analysis using Cytoscape’s MetScape plugin revealed four significantly impacted targets—PTGS2, AKT1, SRC, and MPO—which may play crucial roles in KPF’s therapeutic effects against T1DM (Figure 3D).

To investigate interactions between KPF and key targets, molecular docking was performed using AutoDock Vina. The docking affinities of KPF with four key targets (AKT1, PTGS2, MPO, and SRC) were all greater than −5 kcal/mol, indicating high binding affinity (Figure 3E,F).

### 3.4. Effect of KPF on Caspase-9 Expression and ROS Levels in INS-1 Cells

**Figure 4:**
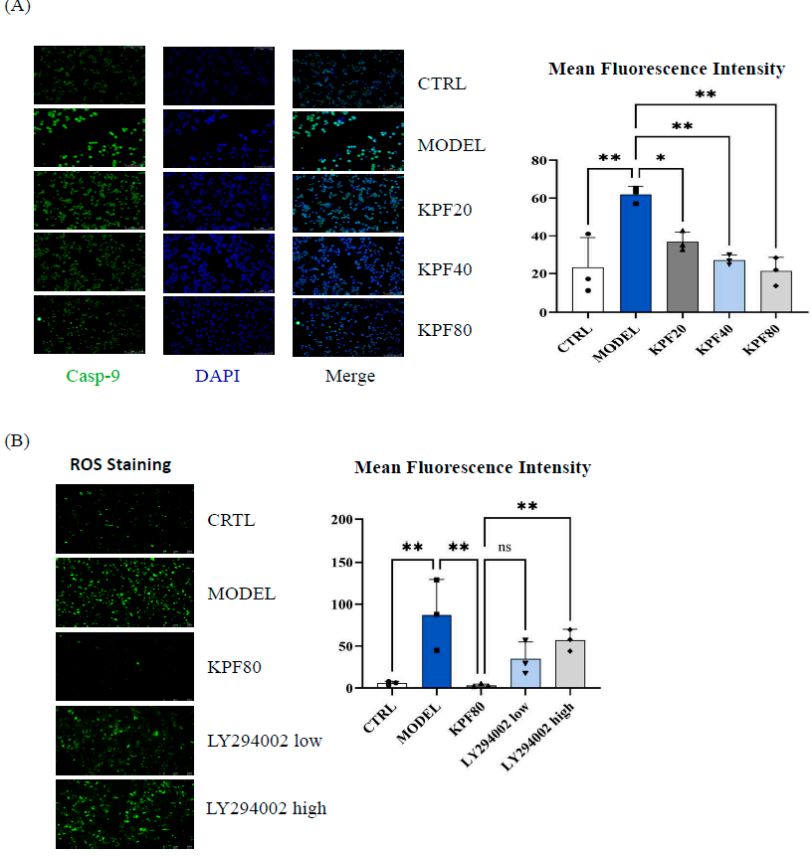
Effect of KPF on caspase-9 expression and AKT-mediated ROS levels in INS-1 cells. (**A**) Immunofluorescence staining of caspase-9 in INS-1 cells after STZ stimulation and KPF treatment. (**B**) ROS levels in INS-1 cells after treatment with KPF and PI3K-AKT pathway inhibitor LY294002. Data are presented as mean ± SD. * *p* < 0.05, ** *p* < 0.01 compared to the model group; ns indicates non-significance.

To evaluate the effect of KPF on the cellular model, immunofluorescence staining was used to detect caspase-9 expression in INS-1 cells. Following STZ stimulation, the model group exhibited significantly increased caspase-9 expression compared to the control group (*p* < 0.01). Treatment with different doses of KPF resulted in a dose-dependent decrease in caspase-9 levels, with a significant reduction in the KPF20 group (*p* < 0.05) and a more pronounced reduction in the KPF40 and KPF80 groups (*p* < 0.01) (Figure 4A).

To determine whether KPF treatment involves the AKT1 pathway, a PI3K-AKT inhibitor (LY294002) was included. ROS levels were measured to assess oxidative stress. The model group had significantly elevated ROS levels compared to the control group (*p* < 0.01). KPF treatment significantly reduced ROS levels (*p* < 0.01). When the pathway inhibitor LY294002 was added, ROS levels significantly increased in the LY294002 high dose group (*p* < 0.01), while the LY294002 low group showed an increase without statistical significance (*p* > 0.05) (Figure 4B).

## 4. Discussion

This study evaluated the protective effects of KPF on metabolic disturbances and pancreatic damage in T1DM mice. Our findings demonstrate that KPF not only improves glycemic control and lipid profiles but also preserves pancreatic β-cell function while modulating key metabolic pathways implicated in T1DM. These findings complement prior research and contribute comparative insights into the multifaceted therapeutic potential of KPF.

KPF treatment notably reduced blood GLU levels and improved lipid parameters by decreasing TG levels and increasing HDL levels in T1DM mice (Figure 1A–F). These findings align with previous studies where KPF ameliorated glucose and lipid metabolism disorders by modulating signaling pathways such as PI3K/AKT and AMPK [12,16,19,20]. Histological analysis showed that KPF preserves pancreatic islet architecture and increases β-cell numbers (Figure 1G,H). Comparable to earlier findings in STZ-induced rats, where insulin immunostaining in pancreatic β-cells was diminished compared to normal controls [21], this study highlights KPF’s role in counteracting β-cell loss. Our findings suggest that KPF treatment counteracts this effect by preserving β-cell numbers and islet structure. This protective effect may be due to KPF’s anti-inflammatory and antioxidant properties, which mitigate oxidative stress and apoptosis in pancreatic tissues [22,23].

Metabolomics profiling identified 94 significantly altered endogenous metabolites between the T1DM and T1DM + KPF50 groups (Figure 2C, Table 1). Notably, KPF modulated levels of 5,10-methylene-tetrahydrofolate (5,10-methylene-THF), a critical intermediate in folate and one-carbon metabolism essential for DNA synthesis and repair [24,25]. This finding aligns with the broader literature emphasizing the importance of folate metabolism in cellular function and survival. By influencing 5,10-methylene-THF levels, KPF may enhance nucleotide biosynthesis and β-cell survival. Additionally, KPF reduced palmitoylcarnitine levels, a metabolite associated with mitochondrial dysfunction and insulin resistance in diabetes [26,27,28]. This reduction suggests enhanced fatty acid oxidation and mitochondrial function, corroborating earlier studies linking palmitoylcarnitine modulation with improved insulin sensitivity.

The alteration of triphosphate metabolites, vital for energy transfer and nucleotide synthesis, indicates that KPF may improve cellular energy homeostasis [29,30]. Moreover, modulation of vasopressin levels by KPF could contribute to improved vascular function and glycemic control, as vasopressin is linked to glucose metabolism and diabetic complications [31]. Network pharmacology analysis highlighted the PI3K/AKT/mTOR signaling pathway as a key mechanism underlying KPF’s effects (Figure 3B,C, Table 2). Molecular docking showed high binding affinities of KPF with targets such as AKT1, suggesting direct interactions that modulate this pathway (Figure 3E,F). These findings support previous research indicating the role of the PI3K/AKT pathway in enhancing β-cell survival and improving insulin signaling.

In vitro, KPF ROS production and caspase-9 expression in INS-1 cells lead to decreased apoptosis (Figure 4A). The increase in ROS levels upon addition of the PI3K-AKT inhibitor LY294002 confirms that KPF’s antioxidant effects are mediated through the PI3K/AKT/mTOR pathway (Figure 4B) [14,15,16].

This study is the first to systematically elucidate KPF’s multi-target and multi-pathway mechanisms in a T1DM model, extending its potential therapeutic applications beyond Type 2 diabetes. Although promising, these findings are based on animal models; thus, clinical trials are needed to assess KPF’s safety and efficacy in humans. Future research should explore KPF’s synergistic effects with existing antidiabetic drugs and further investigate its molecular mechanisms, particularly regarding the PI3K/AKT/mTOR pathway.

## 5. Conclusions

KPF exhibits significant protective effects against T1DM-induced metabolic disturbances and pancreatic damage. By improving glucose and lipid metabolism, preserving β-cell function, and modulating key pathways, KPF holds potential as a therapeutic agent for T1DM management.

## Figures and Tables

**Table 1:** Major differential metabolites between T1DM and T1DM + KPF50 groups.

No.	m/z	Compound Names	ESI Mode	HMDB ID	PubChem ID	KEGG ID	log_2_(FC)	−log_10_p	AUC
1	499.2027	5,10-Methylene-THF	positive	HMDB0001533	439175	C00143	1.82	4.23	1.00
2	442.3735	Palmitoylcarnitine	positive	HMDB0000222	11953816	C02990	7.07	3.00	1.00
3	364.0869	5-Thymidylic acid	positive	HMDB0001227	9700	C00364	−1.22	4.33	1.00
4	348.0586	Uridine 2′,3′-cyclic phosphate	positive	HMDB0011640	439715	C02355	−3.09	3.21	1.00
5	258.9135	Triphosphate	positive	HMDB0003379	983	C00536	1.18	4.06	1.00
6	239.1140	Anserine	negative	HMDB0000194	112072	C01262	3.97	4.48	1.00
7	175.0043	Phenol sulphate	positive	HMDB0060015	74426	C02180	1.64	6.96	1.00
8	159.0201	Cysteinylglycine	negtive	HMDB0000078	439498	C01419	−9.07	7.74	1.00
9	221.0051	L-Cystine	negative	HMDB0000192	67678	C00491	-4.89	2.04	0.98
10	1056.4262	Vasopressin	positive	HMDB0001980	53477739	C00840	−3.56	2.10	0.97
11	165.1261	Undecylenic acid	negative	HMDB0033724	5634	C13910	1.28	3.61	0.97
12	247.0476	Pyridoxamine 5′-phosphate	negative	HMDB0001555	1053	C00647	1.78	2.84	0.93
13	721.1320	UDP-N-acetylmuraminate	positive	HMDB0011720	1169	C01050	−1.37	1.93	0.92
14	426.3730	Vitamin D3	positive	HMDB0000876	5280795	C05443	−2.27	2.94	0.92
15	338.1617	Didemethylcitalopram	positive	HMDB0060472	162976	C16609	−1.37	2.12	0.92
16	293.1341	Deoxyadenosine	positive	HMDB0000101	13730	C00559	−1.58	2.77	0.92
17	291.1355	Argininosuccinic acid	positive	HMDB0000052	16950	C03406	3.16	2.16	0.92
18	173.0552	Formiminoglutamic acid	negative	HMDB0000854	439233	C00439	1.03	2.17	0.92
19	225.0795	Sinapic acid	positive	HMDB0032616	637775	C00482	−3.07	2.58	0.90
20	389.2767	Cholic acid	negative	HMDB0000619	221493	C00695	1.07	1.74	0.87
21	307.2592	Dihomo-gamma-linolenic acid	positive	HMDB0002925	5280581	C03242	−2.07	1.95	0.85
22	383.9977	CDP	negative	HMDB0001546	6132	C00112	1.77	1.32	0.83
23	203.0523	L-Cystathionine	negative	HMDB0000099	439258	C02291	2.84	1.59	0.82

Significantly differential metabolites between T1DM and T1DM + KPF50 groups. This table lists the key metabolites that were significantly altered between the T1DM and T1DM + KPF50 groups. These metabolites were identified using biomarker analysis and are potential indicators of the therapeutic response to KPF treatment. Data include metabolite names, m/z values, ESI mode, relevant database identifiers (HMDB, PubChem, KEGG), and statistical parameters such as fold change (log_2_(FC)), *p*-value, and area under the curve (AUC) score.

**Table 2:** Top 10 enriched pathways for core targets in network pharmacology analysis.

Pathway Name	*p*-Value	FDR
Negative regulation of the PI3K/AKT network	6.85 × 10^−6^	0.0101
PI5P, PP2A, and IER3 regulate PI3K/AKT Signaling.	2.63 × 10^−5^	0.0194
Constitutive Signaling by Aberrant PI3K in Cancer	5.11 × 10^−5^	0.0252
Interleukin-4 and Interleukin-13 signaling	1.29 × 10^−4^	0.0474
PI3K/AKT Signaling in Cancer	3.80 × 10^−4^	0.1121
Cysteine formation from homocysteine	8.05 × 10^−4^	0.1618
Abacavir transmembrane transport	8.05 × 10^−4^	0.1618
Metabolism of folate and pterines	8.79 × 10^−4^	0.1618
Interleukin-18 signaling	1.56 × 10^−3^	0.2560
CTLA4 inhibitory signaling	2.29 × 10^−3^	0.3364

This table summarizes the top 10 pathways enriched for core targets identified through network pharmacology analysis of KPF treatment in T1DM mice. These pathways provide insights into the mechanisms underlying KPF’s therapeutic effects, including pathways related to PI3K/AKT signaling, interleukin signaling, and folate metabolism. Data include pathway names, *p*-values, and false discovery rates (FDR).

## Data Availability

The original contributions presented in this study are included in the article/Supplementary Materials. Further inquiries can be directed to the corresponding author.

## Supplementary Materials

The following supporting information can be downloaded at: https://www.mdpi.com/article/10.3390/foods13233797/s1, Table S1, All Ions (positive mode); Table S2, All Ions (negative mode); Table S3, Biomarker analysis (ROC); Table S4, Core Targets associated pathways; Table S5, Enrichment analysis; Table S6, Metabolites and genes interaction network.
